# Invasive Breast Carcinoma Arising in a Nipple Adenoma After 15 Years: Report of a Rare Case and Literature Review

**DOI:** 10.7759/cureus.8586

**Published:** 2020-06-12

**Authors:** Muhammad Abdulwaasey, Muhammad Usman Tariq, Khurram Minhas, Naila Kayani

**Affiliations:** 1 Histopathology, Pathology and Laboratory Medicine, Aga Khan University Hospital, Karachi, PAK; 2 Histopathology, Aga Khan University Hospital, Karachi, PAK; 3 Pathology and Laboratory Medicine/Histopathology, Aga Khan University Hospital, Karachi, PAK; 4 Pathology and Microbiology, Aga Khan University Hospital, Karachi, PAK

**Keywords:** nipple adenoma, breast cancer, paget's disease, nipple discharge

## Abstract

Nipple adenoma (NA) is a rare benign breast neoplasm that seldom co-exists with breast carcinoma (BC). Majority of these BC are separate from NA, and their origin from NA is an extremely rare event. We herein describe a case of 65-year-old female who had a painless lump for 15 years which increased in size and ulcerated for last six months. Microscopic examination of the wedge biopsy of nipple showed features of NA at superficial aspect and invasive carcinoma from it at the deeper aspect. The patient underwent mastectomy and axillary clearance, which revealed a 4-cm invasive breast carcinoma, no special type with axillary lymph node involvement. The patient received adjuvant chemotherapy, radiotherapy and adjuvant hormonal therapy. The patient is alive and disease-free after 36 months.

NA should be carefully evaluated for co-existent BC because it completely changes the treatment plan and prognosis.

## Introduction

Nipple adenoma (NA) is an uncommon benign breast neoplasm, involving lactiferous ducts orifices, surrounding stroma and overlying epidermis of nipple [1,2]. It most commonly occurs in fifth decade of life and affects both genders [1]. It presents as a palpable mass with nipple distortion and skin changes, such as erythema, thickening, erosion, ulceration and nipple discharge [3-5]. Ultrasound is the most clinically useful imaging modality on which NA appears as a well-defined hypoechoic mass. Mammogram and magnetic resonance imaging are also used for radiological assessment [2,6]. Histologically, tumor shows proliferation of benign ductal and glandular structures, involving the nipple and lactiferous ducts. Variable combinations of sclerosing adenosis, papillomatosis and hyperplastic changes are observed [1,7,8]. Complete surgical resection is the only treatment option [2]. Recurrence is observed in incompletely excised tumors [2].

A small proportion of NA also presents with co-existent ductal carcinoma in situ (DCIS) and/or invasive carcinoma, which alters the treatment and prognosis of these tumors [4,5,7-11]. We herein describe the clinicopathological features of an invasive carcinoma arising from NA. We also reviewed the literature for NA cases with co-existent carcinoma. 

## Case presentation

**Figure 1:**
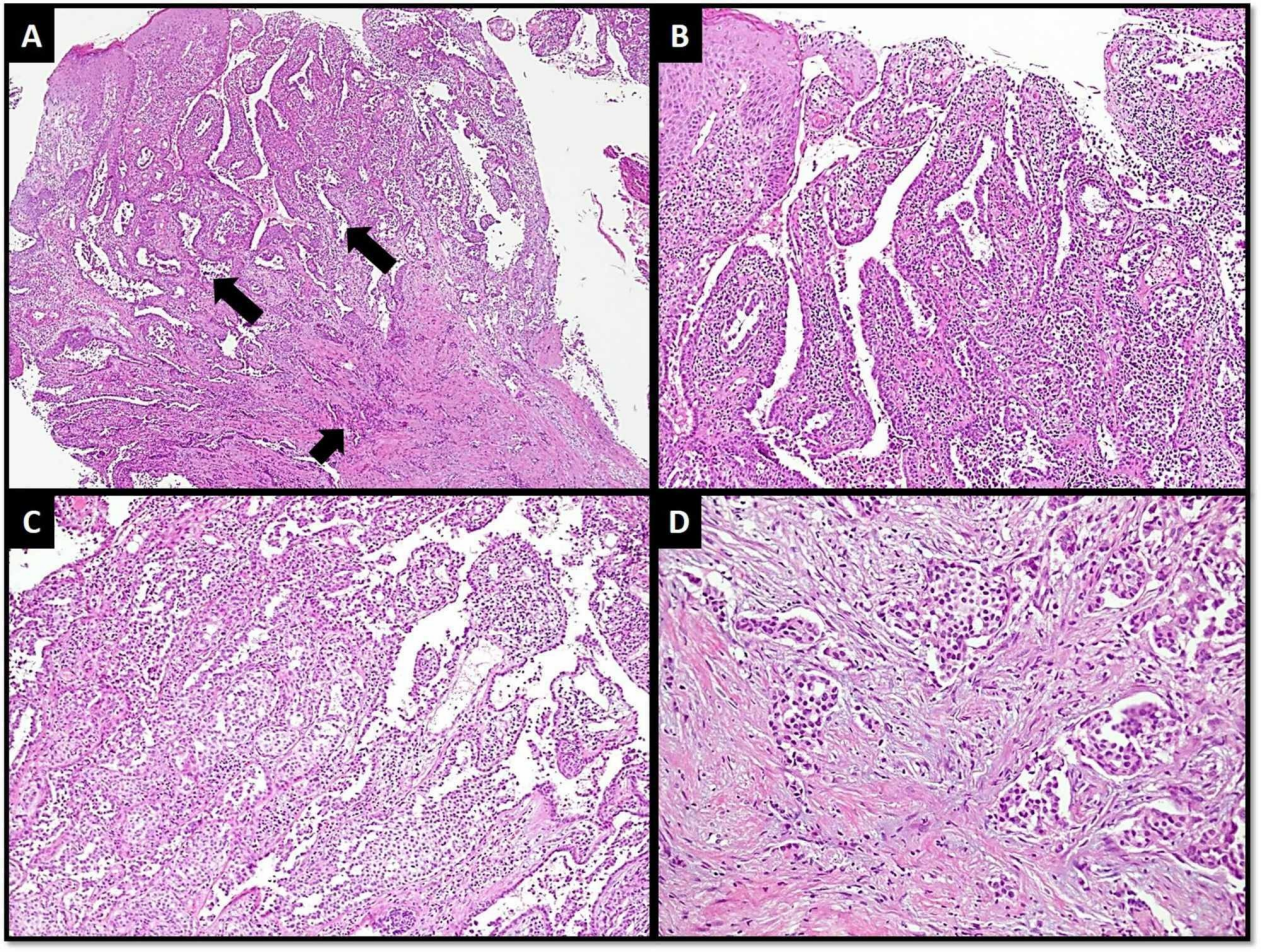
(A) Low-power view of tumor showing features of nipple superficially (larger arrows) and invasive carcinoma at deeper aspect (shorter arrow). (B) Papillomatous pattern in NA. (C) Hyperplastic changes in NA. (D) Nest and tubules of invasive carcinoma. NA, Nipple adenoma

A 65-year-old female presented with a history of painless right subareolar mass for 15 years. The mass became painful for last three years and it gradually increased in size with nipple ulceration for last six months. On physical examination, the nipple was ulcerated with surrounding erythema. A firm subareolar mass and axillary lymph nodes were palpable. Ultrasonogram showed a hypoechoic lesion in subareolar/retroareolar region measuring 3.5 x 3 x 2.4 cm. Mammogram also revealed a spiculated mass. Clinical diagnosis was Paget’s disease and associated malignancy. Wedge biopsy was performed. Microscopically, superficial aspect showed multiple distended lactiferous ducts filled with proliferating epithelial cells showing papillomatosis and hyperplastic changes. These cells showed mild atypia and occasional mitoses. Deeper aspect showed haphazard nests, trabeculae and tubules of neoplastic cells against fibrous background stroma (Figure 1).

**Figure 2:**
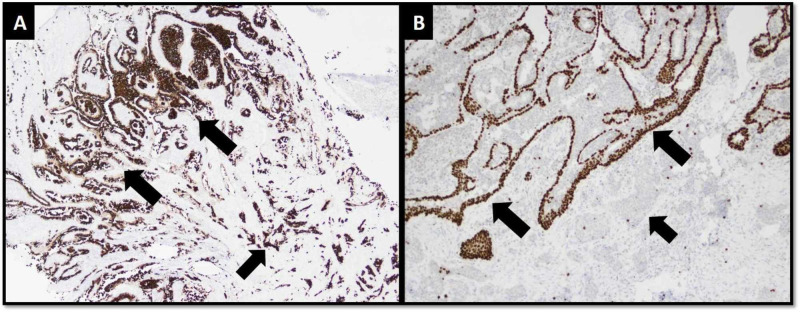
IHC stains. (A) Cytokeratin CAM5.2 highlights papillomatous and hyperplastic nature of NA superficially (larger arrows) and infiltrative pattern of carcinoma at deeper aspect (short arrow). (B) p63 highlights intact myoepithelial cell layer around ducts of NA (larger arrows) and loss of myoepithelial cell layer around invasive carcinoma (short arrow). IHC, immunohistochemistry; NA, nipple adenoma.

Paget's cells were also appreciated in the overlying dermis. An intact myoepithelial cell layer was present around these ducts at the superficial aspect while it was absent around nests and tubules at the deeper aspect (Figure 2).

The diagnosis of “invasive breast carcinoma, no special type (NST) arising in the background of nipple adenoma” was made.

The patient underwent subsequent mastectomy and axillary clearance. Grossly, a gray white firm infiltrating lesion measuring 4 x 3.5 x 2.2 cm was identified beneath nipple and areola. The rest of the breast was unremarkable. Microscopic examination revealed “invasive breast carcinoma, no special type (NST) grade II”. Focal residual NA and Paget’s disease were also appreciated. Two out of 18 lymph nodes were involved by metastatic carcinoma.

The patient received adjuvant chemotherapy, radiotherapy and hormonal therapy. The patient is alive and disease-free after 36 months of follow-up.

## Discussion

NA has been reported in 1.2% cases of breast carcinoma (BC), who underwent mastectomy [7]. Another large review of 224 NA cases reported co-existent BC in 37 (16.5%) cases. In 19 of these cases, carcinoma was ipsilateral, coincidental and separate from NA [7]. Involvement of contralateral breast by invasive carcinoma has also been rarely reported [4]. The invasive carcinoma is of ductal type in vast majority and lobular type has occasionally been reported [7,10]. Over a period of last 35 years, 75 cases of NA had been diagnosed at our institution [12]. Three (4%) cases had co-existent invasive carcinoma (of ductal type). Besides the case described in this report, one of the patients had ipsilateral invasive carcinoma and extensive DCIS which was separate from NA. The other patient had contralateral invasive carcinoma and extensive DCIS.

Koerner comprehensively summarized the findings of 15 cases which were reported in different studies as “in situ or invasive carcinoma arising directly from florid papillomatosis/nipple adenoma” [7]. Eight patients had invasive ductal carcinoma (IDC), two had IDC and DCIS, and two had DCIS only [7]. In one of the cases, synchronous IDC in other quadrant of ipsilateral breast was identified. In another case of DCIS arising in NA, DCIS was also appreciated in contralateral breast [7]. In three cases, Koerner did not find sufficient evidence of NA [7]. In males, almost half of the NAs are associated with carcinoma and this can be attributed to the subareolar origin of majority of BC in males. The origin of BC from NA raises the possibility of precancerous nature of NA [7]. Clinical presentation and disease course in our case also favors this possibility since the lesion (NA) remained asymptomatic for years, as long as it was benign. The changes developed recently, most likely with malignant transformation.

A recent study reported PIK3CA activating mutations in more than 50% cases of NA. Although these mutations are observed in approximately 30% BC, this does not imply a causal relationship since these mutations are more frequently observed in other benign proliferative breast lesions [13].

Four reports of in situ and invasive carcinoma co-existing with NA were further published in the literature [8-11]. Lee et al. reported a case exhibiting three foci of multicentric BC, all separate from NA. One of these tumors was invasive lobular carcinoma, second showed mixed features of ductal and lobular carcinoma while the morphology of the smallest lesion was not described [10]. Sasi et al. reported bilateral NAs. DCIS with microinvasion was appreciated unilaterally [9]. In a series of 13 NA cases, Di Bonito et al. identified DCIS in only a single case [8]. Zhao et al. reported a case of NA co-existing and intraductal papillary carcinoma [11]. 

Histological diagnosis of carcinoma arising in NA can be challenging because of complex architectural patterns observed in NA, such as comedonecrosis, cribriform and micropapillary patterns. Focal necrosis, cytologic atypia and mitotic figures are not common. Sclerosing papillomatous pattern can resemble pseudo-invasion and the picture gets complicated further with focal loss of myoepithelial markers [7,8]. Immunohistochemical (IHC) markers are helpful in highlighting myoepithelial cell layer around majority of the ducts and tubules of NA [7-9]. Careful sampling and examination of the deeper aspect of the lesion should be done to rule out the presence of invasive carcinoma. A panel of IHC markers should be applied to assess the presence/absence of myoepithelial cell layer. It should also be kept in mind that the invasive carcinoma can arise from larger lactiferous ducts (not harboring NA) [7,14].

Paget’s disease in the overlying dermis is commonly observed and it is considered a reliable indicator of malignancy arising in NA. IHC markers cytokeratin CAM5.2 and CK7 are helpful in highlighting Paget's cells [7]. In cases without concomitant Paget’s disease and invasive carcinoma, diagnosis of “DCIS arising in NA” should be made with great caution [7]. 

After complete removal of NA, the risk of subsequent carcinoma development is low [4]. However, in situ and invasive carcinoma have been reported to occur in three patients, 3-17 years after complete excision of NA [7]. Therefore, clinical follow-up is a wise approach [4].

## Conclusions

We conclude that co-existence of NA with BC is an extremely rare event of prime importance. It should always be kept in mind and carefully searched for during histological evaluation of nipple biopsy specimens.
